# Mutation distributions among patients with congenital adrenal hyperplasia from five regions of Brazil: a systematic review

**DOI:** 10.20945/2359-3997000000593

**Published:** 2023-01-25

**Authors:** Silvério S. Hosomi, Igor C. Salles, Tânia A. S. S. Bachega

**Affiliations:** 1 Universidade de São Paulo Faculdade de Medicina São Paulo SP Brasil Faculdade de Medicina, Universidade de São Paulo, São Paulo, SP, Brasil.; 2 Universidade de São Paulo Faculdade de Medicina Hospital das Clínicas, Disciplina de Endocrinologia e Metabologia São Paulo SP Brasil Unidade de Adrenal, Laboratório de Hormônios e Genética Molecular/ LIM42, Hospital das Clínicas, Disciplina de Endocrinologia e Metabologia, Faculdade de Medicina, Universidade de São Paulo, São Paulo, SP, Brasil.

**Keywords:** Congenital adrenal hyperplasia, *CYP21A2* gene, Brazilian patients, molecular diagnosis, classical forms, 21-hydroxylase deficiency

## Abstract

Congenital adrenal hyperplasia due to 21-hydroxylase deficiency is an autosomal recessive disorder caused by *CYP21A2* gene mutations, and its molecular diagnosis is widely used in clinical practice to confirm the hormonal diagnosis. Hence, considering the miscegenation of the Brazilian population, it is important to determine a mutations panel to optimise the molecular diagnosis. The objective was to review the *CYP21A2* mutations’ distribution among Brazilian regions.Two reviewers screened Brazilian papers up to February 2020 in five databases. The pair-wise comparison test and Holm method were used in the statistical analysis. Nine studies were selected, comprising 769 patients from all regions. Low proportion of males and salt-wasters was identified in the North and Northeast regions, although without significant difference. Large gene rearrangements also had a low frequency, except in the Center-West and South regions (p < 0.05). The most frequent mutations were p.I172N, IVS2-13A/C>G, p.V281L and p.Q318X, and significant differences in their distributions were found: p.V281L was more frequent in the Southeast and p.Q318X in the Center-West and Northeast regions (p < 0.05). Thirteen new mutations were identified in 3.8%-15.2% of alleles, being more prevalent in the North region, and six mutations presented a founder effect gene. Genotype-phenotype correlation varied from 75.9%-97.3% among regions. The low prevalence of the salt-wasting form, affected males and severe mutations in some regions indicated pitfalls in the clinical diagnosis. The good genotype-phenotype correlation confirms the usefulness of molecular diagnosis; however, the Brazilian population also presents significant prevalence of novel mutations, which should be considered for a molecular panel.

## INTRODUCTION

Congenital adrenal hyperplasia (CAH) is an autosomal recessive disease and a common inborn error of metabolism. The disease is characterized by enzymatic deficiencies affecting cortisol secretion, leading to an overproduction of adrenocorticotropic hormone (ACTH). Excessive ACTH overstimulates steroidogenesis in the adrenal cortex. CAH is most commonly caused by 21-hydroxylase deficiency (21OHD), representing 90%-95% of all cases (1). Patients develop different clinical manifestations depending on residual 21-hydroxylase activity. CAH is traditionally classified into classical and nonclassical (NC) forms. The classical form is subdivided into simple virilizing (SV) and salt-wasting (SW) forms. In the former, residual enzymatic activity is typically between 3% and 7%. Patients with the SV form present with signs of prenatal and postnatal hyperandrogenism, such as prenatal external genitalia virilization in females, pseudo-precocious puberty, increased growth rate, and advanced bone-age that results in short stature. Residual enzymatic activity in the SW form is less than 2%. Patients present with hyperandrogenic symptoms similar to patients with SV, and show severe impairment of aldosterone secretion. Thus, hyponatremic dehydration occurs during the neonatal period. Residual enzymatic activity in the nonclassical form is greater than 20%, and patients present with late-onset hyperandrogenic manifestations (2). Clinical forms are diagnosed by increased 17-OH progesterone (17-OHP) levels, typically higher than 50 ng/mL and 10 ng/mL in classical and nonclassical forms, respectively (3, 4). The incidence of classical forms varies from 1:10,000 to 1:18,000 live births, whereas the nonclassical form has a higher incidence, affecting approximately 1:1000 neonates (4–8).

The gene that encodes 21-hydroxylase, *CYP21A2*, is located on the short arm of chromosome 6 in tandem with its highly homologous pseudogene, *CYP21A1P*. The latter gene is not translated due to the presence of several mutations. The high homology of these genes facilitates unequal crossover during meiosis. Fragments from the pseudogene are transferred to the active gene. This mechanism is the primary source of mutations in 21OHD. To date, approximately 300 *CYP21A2* mutations are described in several populations, mostly originating from conversion events involving the pseudogene (5,9,10).

Further, a good correlation is reported between residual enzymatic activity *in vitro* and clinical manifestations for *CYP21A2* mutations (1). Most CAH patients are compound heterozygotes, and clinical form correlates with the allele carrying the mutation that supports higher enzyme activity (2,5,11). This genotype-phenotype correlation is useful in clinical practice to predict clinical form, especially for prenatal diagnosis and neonatal screening (12–15). Still, a French study indicates that neonatal screening using serum 17-OHP levels has a low positive predictive value in special situations, such as in premature infants. That is, a significant false positives rate is observed, causing increased workload for health care systems and concern among parents (16). Thus, in special situations, genetic analysis of *CYP21A2* can be useful clinically as a confirmatory test.

The incidence of *CYP21A2* mutations might vary among ethnic groups; previous studies observed a lower frequency of *CYP21A2* deletions in the Brazilian population than in Caucasian subjects (17). Interestingly, a higher frequency of mutations in the founder effect gene, not derived from the pseudogene, was identified in the former individuals (18, 19). Our study collected data on *CYP21A2* mutations in five Brazilian regions to identify differences in prevalence. We thus provide a better understanding of regional and national genetic variation and contribute information to support future healthcare policies.

## METHODS

The Preferred Reporting Items for Systematic Reviews and Meta-Analyses (PRISMA) 2020 checklist and flow diagram were used to support the study (20). This information is available in supplementary material (S1). This review was not registered at the beginning of the study. However, the methodology was not modified during the study.

### Search strategy and information sources

A search of five databases was conducted for publications up to April 2020: the Brazilian Digital Library of Theses and Dissertations (BDTD), Embase, Latin American and Caribbean Health Sciences Literature (LILACS**)**, PubMed, and the Scientific Electronic Library Online (SciELO). The following terms of controlled vocabulary (MeSH) or their alternatives were used in each database: “Cytochrome P450 Family 21”, “Congenital, Adrenal Hyperplasia”, “Brazil”, and “Steroid 21-Hydroxylase”. Controlled vocabulary terms and their synonyms helped maximize retrieval. Literature was later updated for studies published from 2020 to 2022 using the same databases and terms. The full search strategy is available in supplementary material (S2).

### Eligibility criteria

Observational studies that analyzed *CYP21A2* mutations in Brazilian patients with CAH were eligible for initial screening. Studies or reviews that reported on genotyping of non-Brazilian populations were excluded.

### Selection process

Two reviewers worked independently in the selection process, which consisted of an initial title and abstract screening, followed by a full-text screening. The studies not eliminated in this first stage were fully assessed by reviewers to select studies that fulfilled the selection criteria. Reasons for exclusions were provided in written form. Disagreements were discussed and resolved between the reviewers. When an agreement could not be reached, a third reviewer was used to settle the matter.

### Data collection and data items

Data extraction was performed by one reviewer and verified by the other. Numbers of participants, sex, Brazilian region, method of genotype analysis, frequency of mutations, clinical presentation, genotype groups and genotype-phenotype correlations were extracted.

The number of alleles with respective variants were divided by the number of all unrelated alleles belonging to the correspondent region to assess variant contribution to the regional *CYP21A2* mutation panel. If studies included data from the same cohort or whenever it was not possible to determine whether different studies from the same region included the same patients, only the study with the higher number of patients was used.

Patients from selected studies had their genotype classified according to Krone and Arlt, 2009 (21). Patients with the Null genotype carry mutations in both alleles that predict total impairment of enzymatic activity, patients with the A genotype carry the I2 splice mutation (<2% activity), homozygous or compound heterozygous with variants from Null group. Patients in B group carry at least one mutation that predicts a residual enzyme activity between 3% and 7%, homozygous or compound heterozygous with the former mutations. Similarly, patients from group C carry mutations that predict 20%-60% residual enzymatic activity, homozygous or compound heterozygous with the former mutations. Patients from group D carry mutations for which impairment was not evaluated, and patients from group E carry only one allele with an identified pathogenic variant.

### Bias risk assessment

Two reviewers worked independently in the bias risk assessment process, using the Study Quality Assessment Tool developed by the National Heart, Lung, and Blood Institute (Study Quality Assessment Tools – NHLBI, NIH, 2022). After using the questionnaire for cross- sectional studies, the articles were categorized into three risk groups: good, fair, and poor. Disagreements were discussed and resolved between the reviewers. When an agreement could not be reached, a third reviewer was used to settle the matter.

### Effect measures and synthesis methods

Comparisons of frequencies from each mutation in different regions used pairwise comparisons with exact binomial tests. Differences in the proportion of clinical forms and sex ratios used tests of multiple comparisons of proportions with correction for *p*-adjusted values (22). Significance was defined as *p* < 0.05. All analyses used R statistical software, version 4.0.5 (23).

## RESULTS

The initial literature search retrieved 956 records; 32 were duplicates. Reviewers independently screened 924 unique records by title and abstract, and 864 were excluded. Among the last 60 records, nine were included for quantitative analysis after full-text screening. Other studies were excluded because of small sample sizes that overlapped with a major cohort in other studies. The search update retrieved 77 records; 15 were duplicates. Two reviewers independently screened 62 unique records by title and abstract, but all were excluded using the same inclusion and exclusion criteria used in the initial search.

**Figure 1:**
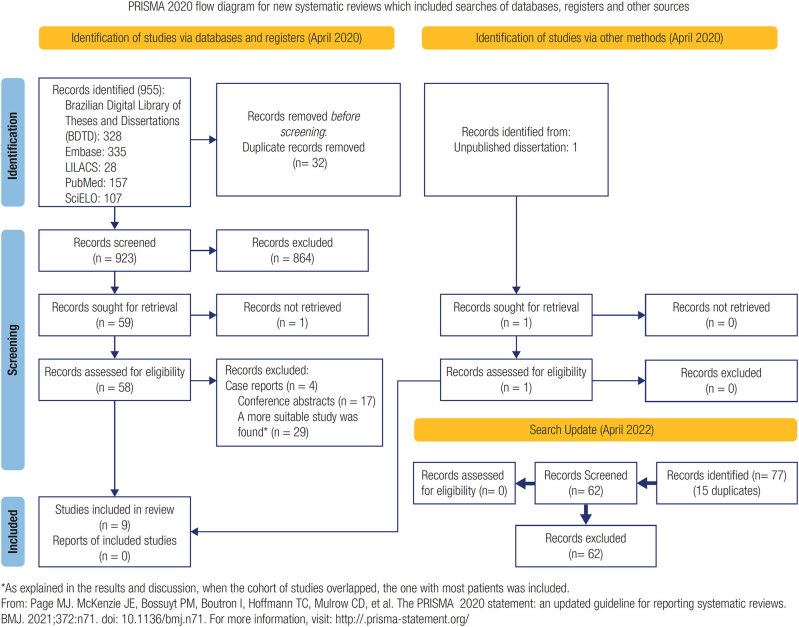
Flow diagram

**Figure 2:**
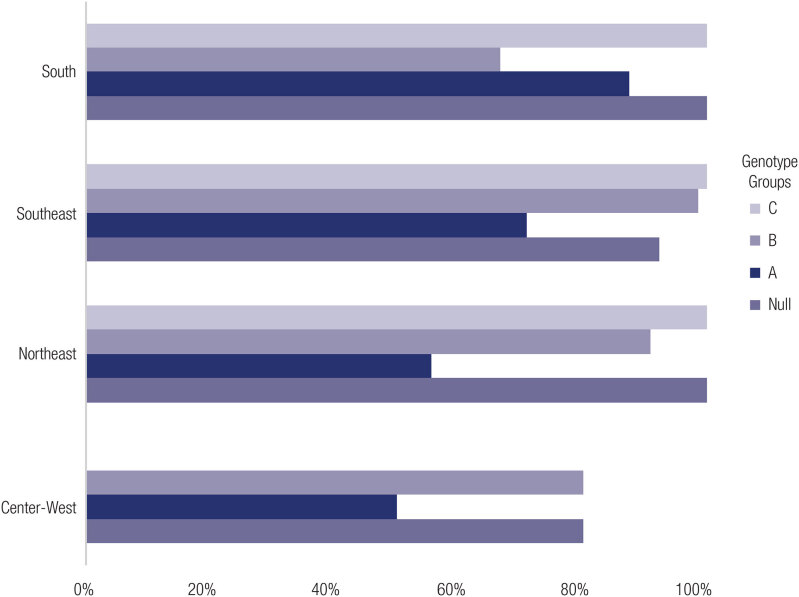
Proportion of genotypes Null and A in the salt wasting patients, and B and C between simple virilizing and nonclassical patients.

The PRISMA flow diagram (Figure 1) summarizes the literature search. Numbers of articles found per region were one from the North region (24), two from the Northeast (25, 26), one from the Central West (27), one from the South (28), and four from the Southeast (29–32). Notably, only neonatal screening studies were available from the South region. Overall, the bias risk assessment indicated good quality for the studies selected, except for the North region article. The methodology for this study lacked the precision to discriminate the prevalence of pathogenic variants, thus increasing measurement bias risk. In total, 1,395 unrelated alleles from 769 patients were analyzed, of which the Southeast region accounted for 1,130 (81%) of alleles. Three studies did not include NC patients (26,27,29), and one study did not include classical patients (32). Study cohorts and methodology are summarized in Table 1, and the proportion of CAH patients with classical CAH are displayed in Figure 2.

**Table 1:** Selected studies per Brazilian region and their respective cohorts and the applied methodologies to screen mutations

Author	Region	Number of patients	Large gene rearrangement	Point-mutation
De Carvalho D^28^	Southeast	158 SW/116 SV/206 NC	Southern-Blot, MLPA	Sequencing, AS-PCR
Paulino L^27^	Southeast	27 SW/14 SV	Southern-Blot	AS-PCR
Coeli-Lacchini F^29^	Southeast	32 SW/29 SV/29 NC	MLPA	Sequencing, AS-PCR
Castro P^30^	Southeast	0 SW/0 SV/10 NC	MLPA	AS-PCR
Kopacek C^26^	South	12 SW/3 SV/7 NC	MLPA	SNaPshot assay
Araújo V^25^	Center-West	15 SW/14 SV	MLPA	Sequencing, AS-PCR
Carvalho T^22^	North	15 SW/19 SV/12 NC	-	Sequencing
Toralles M^24^	Northeast	10 SW/20 SV	-	AS-PCR
Campos V^23^	Northeast	7 SW/9 SV/5 NC	-	AS-PCR

Large gene rearrangements: CYP21A2 deletions, large gene conversions; SW: salt wasting; SV: simple virilizing; NC: nonclassical; MLPA: Multiplex Ligation-dependent Probe Amplification.

**Figure 3:**
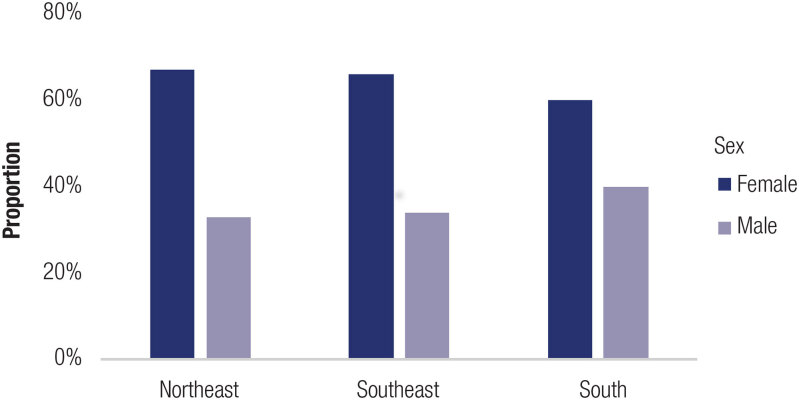
Female and male ratio of CAH patients in the Brazilian regions. The statistical analysis used the test of multiple comparisons for proportion with correction for p-adjusted value by Holm.

It was possible to determine the ratio between females and males according to the clinical form in five studies: one from the South, two from the Northeast, and two from the Southeast region. The female/male ratio between SW and SV forms was not determined in the latter study. The proportion of females (F) and males (M) among SW patients was 7.5:1 (15 females and two males) in the Northeast and 1.4:1 (seven females and five males) in the Southern regions. The F/M ratio among SV patients was 1.2:1 (16 females and 13 males) in the Northeast and 2:1 (two females and one male) in the South. The F/M ratio among classical patients was 1.9:1 (179 females and 95 males) in the Southeast (Figure 3). Significant differences in F/M and SW/SV ratios among Brazilian regions were not observed (*p* > 0.05).

### Large gene rearrangements

Multiplex ligation-dependent probe amplification (MLPA) was used in four studies for detection of large gene rearrangements (26,29,31,32); Southern blotting was used in one (29); another study employed both methods (30). Neither North nor Northeast region patients were screened for large gene rearrangements. The term, large gene rearrangements (LGR), was used whenever a *CYP21A2* deletion or large gene conversion was not specified. In the Southeast region, *CYP21A2* deletions (LD) were identified in 27 (2.4%) of unrelated alleles, and large gene conversions (LGC) in 20 (1.8%) and LGR in 69 (6.1%) of unrelated alleles. In the South region, *CYP21A2* deletions were identified in five (12.5%) of unrelated alleles and LGC in three (7.5%); in the Center-West, *CYP21A2* deletion and LGC were identified in nine (18%) and eight (16%) of unrelated alleles, respectively.

### Point mutations

Detection of microconversion events from the pseudogene was performed in all studies. These studies mainly used Allele-Specific Polymerase Chain Reaction (AS-PCR), while the South region used the SnapShot assay. Regarding the number of mutations screened using site-directed mutations tests, the South region screened 12-point mutations, the Center-West 14, the Northeast 14, the Southeast 14 and the North region applied the entire gene sequencing methodology. Results are displayed in Table 2.

**Table 2:** Frequency (%) of the most common point mutations in the *CYP21A2* gene in CAH patients from the different Brazilian regions according to clinical form

Mutations	Center-West			Northeast			North			Southeast			South		
	SW	SV	NC	SW	SV	NC	SW	SV	NC	SW	SV	NC	SW	SV	NC
IVS2-13A/C>G	10	12	0	7.2	8. 4	2.4	21.7	14.1	3.2	12.4	5.8	3.8	17.5	5	2.5
p.Q318X	14	2	0	10.8	6	0	3.2	4.3	5.4	4.5	2	0.4	2.5	0	5
p.I172N	2	2	0	1.2	10.8	0	0	1.1	1.1	0.6	9.9	0.8	2.5	7.5	0
p.V281L	4	4	0	1.2	1. 2	4.8	3.2	5.4	4.3	0.4	0.2	25.7	0	0	20
p.R356W	0	0	0	2.4	4. 8	1.2	0	1.1	0	3.9	1.8	1	2.5	0	5
p.L307Ffs	4	0	0	3.6	0	0	0	1.1	1,1	1.5	0.2	0.4	2.5	2.5	0
CL6	0	0	0	0	0	0	3.2	3.2	1.1	0.6	0.4	0.2	2.5	0	0
p.G110Vfs	0	0	0	0	0	1.2	0	2.1	1.1	1.3	0.2	0.4	0	0	0
p.P30L	2	2	0	0	0	1.2	0	6.5	1.1	0	0.4	0.9	0	0	0
p.P453S	0	2	0	0	2. 4	0	0	0	0	0	0	1	0	0	5

Data obtained from unrelated alleles. SW: salt wasting; SV: simple virilizing; NC: nonclassical. CL6 correspond to p.I236N, p.V237E and p.M238K mutations.

The three most common mutations in the Center- West region were IVS2-13A/C>G (22% of unrelated alleles), p.Q318X (16%), and p.V281L (8%). In SW and SV patients, the most frequent mutations were p.Q318X (14%) and IVS2-13A/C>G (12%), respectively. In the Northeast region, the most frequent mutations were IVS2-13A/C>G (18% of the unrelated alleles), p.Q318X (16.8%), and p.I172N (12%). The SW form was associated with the p.Q318X mutation (10.8%), SV with p.I172N (10.8%), and NC with p.V281L (4.8%). IVS2-13A/C>G (39%), p.Q318X (5.4%), and p.V281L (12.0%**)** were most common in the North region. The SW form was associated with IVS2-13A/C>G (21.7%), SV with IVS2-13A/C>G (14.1%), and NC with p.Q318X (5.4%). In the Southeast, the p.V281L mutation was identified in 26.3% of unrelated alleles, IVS2-13A/ C>G in 22%, and p.I172N in 11.3%. The SW form was associated with IVS2-13A/C>G (12.4%), SV with p.I172N (9.9%), and NC with p.V281L (25.7%). The IVS2-13A/C>G mutation was identified in 25% of unrelated alleles in the South, with p.V281L in 20%, and p.I172N in 10%. The SW form was associated with IVS2-13A/C>G (21.9%), SV with p.I172N (7.3%), and NC with p.V281L (19.5%).

Regarding the distribution of *CYP21A2* mutations in the Brazilian population, the p.V281L mutation accounted for 23.6% of all alleles. IVS2-13A/C>G accounted for 23%, p.I172N for 10.5%, p.Q318X for 8.3%, p.R356W for 6.3%, and p.P30L and p.L307fs for 2.8% each. Other mutations account for the remaining observations: p.P453S for 2.1%, p.G110Vfs for 1.9%, [p.I236N, p.V237E, p.M238K] designed as CL6 for 1.6%, and LGR for 8.6% of all alleles.

The prevalence of LGR was low in the Brazilian population, yet it represented 34% and 20% of alleles in the Center-West and South regions, respectively. Still, the IVS2-13A/C>G mutation still showed the highest prevalence overall. It was the most common micro-conversion event, except in the Southeast, where it was second most common. In this region, the p.V281L mutation was most prevalent. The p.Q318X mutation was the second most common mutation in the North, Northeast, and Center-West regions, and in the South region, the p.V281L mutation was second in prevalence. The p.P453S mutation was rare in the Center-West and Southeast regions, p.R356W rare in the North, p.P30L and p.G110Vfs in the Northeast, and p.Q318X, p.R356W and p.P453S in the South.

Some differences in *CYP21A2* pathogenic variants among Brazilian regions were found (p < 0.05). A significantly higher frequency of p.Q318X was observed in the Center-West and Northeast regions compared to the South. Further, a lower prevalence of p.V281L was seen compared to the Southeast. Finally, LGR mutations were more common in the Center- West and South relative to other regions.

### Mutations not derived from pseudogene conversion events

Entire gene sequencing methodology was used in studies from three Brazilian regions. In the North, 15.2% of the alleles carried mutations not derived from microconversion events with the pseudogene and the following pathogenic variants were identified: p.R339H, p.G375S and p.R479L. In the Center-West, prevalence was 6% of alleles, and three pathogenic variants were identified: p.R444X, IVS2+5G>A, and p.R479L. Prevalence in the Southeast was 3.8% of alleles, and the pathogenic variants included p.G424S, IVS2-2A>G, p.R408C, p.R426H, p.S170Nfs, p.H365Y, and p.E351V. Among twenty-one novel mutations not derived from the pseudogene, six presented a founder effect gene, including p.G424S, IVS2-2A>G, p.R408C, p.R426H, p.S170Nfs, and p.H365Y.

### Alleles carrying two or more point mutations

Alleles carrying more than one point mutation were detected in approximately 5.9% of all alleles. In the Northeast, South and Southeast regions, multiple mutations accounted for 7.2%, 2.4%, and 5.5% of alleles. In the Center-West, mutation segregation analysis in parent DNA samples was not performed, and frequency was not possible to determine in the North.

### Alleles with no mutation identified

Alleles without identified pathological variants accounted for 5.3% of all alleles, with 25.3% of alleles in the Northeast region, 6% in the Center-West, 2.1% in the Southeast, and 2.1% in the North region.

### Genotype-phenotype correlation

**Figure 4:**
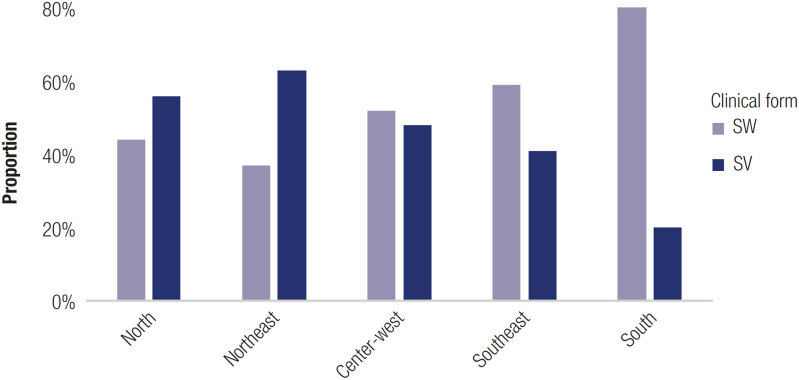
Salt wasting and simple virilizing ratios in all Brazilian regions. SW: salt wasting; SV: simple virilizing.

Data from Brazilian regions typically show good genotype-phenotype correlation, ranging from 90 to 95%, as described in several studies (13,14,33-36). The Southeast region exhibited the highest correlation, 90% to 97.3%, followed by the South (90%), Northeast (81.5%), and Center-West (75.9%) (Figure 4). Data concerning genotype-phenotype correlation were not reported in the single North region study.

Excluding two Southeast and one North region studies, genotypes could be classified according to Krone an cols. (25-29,31,33). The correlation between the Null group, SW form, in the Center- West, Northeast, South, and Southeast regions was 80%, 100%, 92% and 100%, respectively; in the “A” group with the SW form was: 50%, 55.5%, 70.9% and 87.5%; in the B group patients with the SV form reported generally strong correlations of 100%, 91%, 98.5% and 66.7%, respectively. Correlations among C group patients with the NC form in the Northeast, South, and Southeast regions were 75%, 100% and 100%, respectively. Only 0.9% of patients carried the D genotype. Finally, patients carrying E genotype with at least one allele without identified mutations comprised 17.2%, 46%, 7.7% and 0% of patients from the Center-West, Northeast, Southeast and South regions, respectively.

Prevalence of the SV in A group was 50%, 55.5%, 71%, and 87.5% in the Center-West, Northeast, Southeast and South regions, respectively. The SW form comprised the remaining cases. The Southeast region included only one patient carrying the A group genotype that presented with the NC form. Three regions included patients with the E group genotype. Five patients presented with the classical form in the Center-West, 32 presented with the NC form in the Southeast, and 23 with classical and two with NC forms in the Northeast. No significant differences were identified in the prevalence of A and B genotypes among Brazilian regions (p > 0.05).

## DISCUSSION

CAH is a common genetic disorder that can be precociously diagnosed by neonatal screening. Early identification allows prompt treatment and helps prevent severe comorbidity. Generally, filter paper and serum steroid measurements provide good diagnostic efficacy for CAH diagnosis; however, some cases remain with inconclusive diagnosis, and molecular tests can be a useful tool. An adequate molecular panel requires knowledge of the distribution of *CYP21A2* pathogenic variants in the different Brazilian regions. Use of such a panel would support the development of specific strategies that consider the lack of intermarriage among Brazilian subpopulations.

Brazil accepted almost four million immigrants from different parts of the world during the colonial period and continuing to 1933. These immigrants arrived from Italy, Portugal, Spain, Japan, Syria, Lebanon, and other countries. Further, more than five million enslaved Africans entered the country up until the nineteenth century, and indigenous Amerindian populations also contributed to current genetic variability (37–39). These factors led to substantial ethnical diversity, with each region possessing its own genetic profile. To the best of our knowledge, this study is the first to build regional genetic profiles of *CYP21A2* pathogenic variants in Brazil.

The efficacy of clinical diagnosis can be assessed using the ratio of patients with SW and the SV forms of CAH, and by sex ratios. The F:M ratio was not evaluated in studies from Center-West and North regions. In the remaining regions, more females than males presented with classical forms. The F:M ratio did not significantly differ among regions, but, notably, the low ratio of males to females may indicate that males died before being properly diagnosed. Diagnosis of CAH in females is generally facilitated by the presence of atypical genitalia at birth. However, in the South region, where neonatal screening was implemented, the proportion of males to females was slightly but not significantly different from other regions (28). We hypothesize that antenatal use of glucocorticoids to enhance lung maturation before elective cesarean sections, a common but inappropriate practice in Brazil, may increase false-negative results in neonatal screening (5, 40). Significant differences in the proportions of SV and SW forms were not seen among regions. Expected heterogeneity among the five regions in study sample sizes, proportions of clinical forms, and methodologies, including the number of pathogenic variants screened, influenced regional mutation frequencies.

A lower prevalence of LGR was seen, except in the Center-West and South regions, where it was considerably higher. LGR was not screened in studies performed in the North and Northeast regions, which provides a partial explanation for these differences (24–26). *CYP21A2* deletions were associated with classical CAH, and the lower prevalence observed in some regions might suggest that SW neonates were not properly diagnosed in the absence of neonatal screening. Additionally, only patients with the classical forms were studied in the Center-West region (27), consistent with higher prevalence of LGR in comparison to other Brazilian regions. This regional prevalence is similar to the prevalence in Caucasian populations (13, 14).

The Brazilian population displayed some differences in comparison to Caucasian populations. For instance, a higher prevalence of the p.Q318X mutation was reported in the North, Northeast, and Center- West patients (24–27). IVS2-13A/C>G is the most prevalent mutation in various ethnic populations, and is the most frequent pathogenic variant in Brazilian regions, especially among SW patients. IVS2-13A/ C>G and p.I172N are the most frequent pathogenic variants among SV patients, and their distribution was similar among regions. The p.V281L variant was most frequent in the Southeast region and accounted for 25.7% of all mutations screened. This prevalence exceeded IVS2-13A/C>G variant frequency and represents a large proportion of NC patients included in later studies (29–32).

Interestingly, a significant proportion of novel mutations were identified in our Brazilian populations that were not derived from pseudogene conversion events. Novel mutations notable in the North, Center-West, and Southeast regions (24,27,29–32). These findings also reflect the presence of a founder effect gene. We speculate that the higher frequency of mutations with a founder effect gene in some regions represents patients from isolated geographic areas. Since Brazil has substantial ethnic diversity and each state has its own ethnic profile from the colonization period, we could expect subsets of a small population in past decades or centuries to contribute to major genetic drift across the country. In fact, six novel founder effect gene mutations were first described in Brazil: p.G424S, p.R408C, p.H365Y, p.S170Nfs, p.R426H, and IVS2-2A>G (18, 19). Subsequently, p.G424S was also identified in a few countries, such as Portugal, France, and Argentina, the p.R408C was reported in France and Argentina, and p.H365Y was observed in the United Kingdom and the United States (13,41–45). These findings could reflect complex migratory dynamics involving Europe and the Americas.

Studies from the United States and Europe showed that 90%-95% of CAH patient alleles carry identified mutations (34), and these data resulted from the methodologies used to screen mutations. We note similar results in Brazilian regions, above 90% (24,27–32). In contrast, no mutation was identified in approximately one-quarter of alleles in the Northeast region. This finding is likely due to the lower number of mutations screened (25, 26).

Another interesting result is the detection of more than one point mutation per allele. Multiple mutations may cause errors in genotype-phenotype correlations in the absence of mutation segregation analysis in parent DNA samples. The percentage of such alleles was as high as 7.2% in the Northeast region, 2.4% in the South, and 5.5% in the Southeast (25,26,28–32). Studies in the North and Central-West regions did not allow verification of mutation segregation tests. The influence of multiple mutations on genotype-phenotype correlation could not be assessed in these regions.

We did not find significant differences in genotype- phenotype correlations. Genotypes Null and A were associated with the SW form, and whereas B and C with SV and NC forms, respectively. We assume the presence of unidentified mutations (in cis) in the less compromised allele, which could support an SW phenotype in cases of discordant genotype-phenotype correlations. This hypothesis could explain a few cases in the Center-West and Southeast regions. Five patients with classical CAH carried a nonclassical genotype. Entire gene sequencing was not used for these patients, and a severe pathogenic variant was probably not identified. Additionally, some patients carrying genotype A presented with the SV form; however, alternative splicing of the IVS2-13A/C>G mutation (46), leading to higher enzymatic activity, explains the simple virilizing phenotype in these patients. Finally, Brazilian studies showed a good genotype-phenotype correlation; similar findings are also reported for other ethnic groups (13,14,33–36). These findings highlight the utility of molecular tests for neonatal CAH diagnosis and prediction of clinical form in asymptomatic patients. Notably, 17-OHP measurements have been successfully implemented in several countries for cost- effective screening (16, 47). However, limitations of 17-OHP have encouraged the development of other diagnostic methods, including a two-tier approach using other metabolites, such as androstenedione (48). Genetic tests could be an option for complementing neonatal screening diagnosis, considering significant genotype-phenotype correlations.

Our review has a few limitations. Our work was not registered in a formal database of systematic reviews, and the protocol was not made available online. However, we emphasize that methodology was not modified during the execution of the study. Further, we found a different number of studies from each region despite a rigorous search on multiple databases, including theses from national universities. Still, we could not completely overcome this limitation. Finally, patients in different studies from the same region might overlap, leading to an overestimation of the prevalence of some pathogenic variants and clinical forms.

In conclusion, CAH is a frequent and severe genetic disease worldwide. Its molecular pathophysiology is now completely elucidated. In the present study, we examined the distribution of *CYP21A2* pathogenic variants among Brazilian regions. Some different frequencies could be justified by the lower proportion of salt-wasting patients that were not diagnosed due to the absence of neonatal screening. A large proportion of Brazilian patients carried mutations derived from pseudogene conversion events. In contrast, several novel pathogenic variants in the founder effect gene should be considered for a mutation panel. Finally, a significant genotype-phenotype correlation emphasizes the usefulness of molecular diagnosis for clinical application.

## SUPPLEMENTARY MATERIAL

Mutation Distributions among Patients with Congenital Adrenal Hyperplasia from Five Regions of Brazil: A Systematic Review

S1 – PRISMA Checklist 2020

S2 – Full search strategy

**Table:** PRISMA 2020 Checklist

Section and Topic	Item #	Checklist item	Location where item is reported
**TITLE**			
Title	1	Identify the report as a systematic review.	1
**ABSTRACT**			
Abstract	2	See the PRISMA 2020 for Abstracts checklist.	2
**INTRODUCTION**			
Rationale	3	Describe the rationale for the review in the context of existing knowledge.	3
Objectives	4	Provide an explicit statement of the objective(s) or question(s) the review addresses.	4
**METHODS**			
Eligibility **criteria**	5	Specify the inclusion and exclusion criteria for the review and how studies were grouped for the syntheses.	4
**Information** sources	6	Specify all databases, registers, websites, organisations, reference lists and other sources searched or consulted to identify studies. Specify the date when each source was last searched or consulted.	4
**Search** strategy	7	Present the full search strategies for all databases, registers and websites, including any filters and limits used.	4
**Selection** process	8	Specify the methods used to decide whether a study met the inclusion criteria of the review, including how many reviewers screened each record and each report retrieved, whether they worked independently, and if applicable, details of automation tools used in the process.	4
**Data** collection process	9	Specify the methods used to collect data from reports, including how many reviewers collected data from each report, whether they worked independently, any processes for obtaining or confirming data from study investigators, and if applicable, details of automation tools used in the process.	4
Data items	10a	List and define all outcomes for which data were sought. Specify whether all results that were compatible with each outcome domain in each study were sought (e.g. for all measures, time points, analyses), and if not, the methods used to decide which results to collect.	4-5
	10b	List and define all other variables for which data were sought (e.g. participant and intervention characteristics, funding sources). Describe any assumptions made about any missing or unclear information.	5
Study risk of bias assessment	11	Specify the methods used to assess risk of bias in the included studies, including details of the tool(s) used, how many reviewers assessed each study and whether they worked independently, and if applicable, details of automation tools used in the process.	5
Effect measures	12	Specify for each outcome the effect measure(s) (e.g. risk ratio, mean difference) used in the synthesis or presentation of results.	5
Synthesis methods	13a	Describe the processes used to decide which studies were eligible for each synthesis (e.g. tabulating the study intervention characteristics and comparing against the planned groups for each synthesis (item #5)).	5
	13b	Describe any methods required to prepare the data for presentation or synthesis, such as handling of missing summary statistics, or data conversions.	NA
	13c	Describe any methods used to tabulate or visually display results of individual studies and syntheses.	NA
	13d	Describe any methods used to synthesize results and provide a rationale for the choice(s). If meta-analysis was performed, describe the model(s), method(s) to identify the presence and extent of statistical heterogeneity, and software package(s) used.	NA
	13e	Describe any methods used to explore possible causes of heterogeneity among study results (e.g. subgroup analysis, meta-regression).	NA
	13f	Describe any sensitivity analyses conducted to assess robustness of the synthesized results.	NA
Reporting bias assessment	14	Describe any methods used to assess risk of bias due to missing results in a synthesis (arising from reporting biases).	NA
Certainty assessment	15	Describe any methods used to assess certainty (or confidence) in the body of evidence for an outcome.	NA
**RESULTS**			
Study selection	16a	Describe the results of the search and selection process, from the number of records identified in the search to the number of studies included in the review, ideally using a flow diagram.	5
	16b	Cite studies that might appear to meet the inclusion criteria, but which were excluded, and explain why they were excluded.	5
Study characteristics	17	Cite each included study and present its characteristics.	05-Jun
Risk of bias in studies	18	Present assessments of risk of bias for each included study.	05-Jun
Results of individual studies	19	For all outcomes, present, for each study: (a) summary statistics for each group (where appropriate) and (b) an effect estimate and its precision (e.g. confidence/credible interval), ideally using structured tables or plots.	06-Jul
Results of syntheses	20a	For each synthesis, briefly summarise the characteristics and risk of bias among contributing studies.	6
	20b	Present results of all statistical syntheses conducted. If meta-analysis was done, present for each the summary estimate and its precision (e.g. confidence/credible interval) and measures of statistical heterogeneity. If comparing groups, describe the direction of the effect.	NA
	20c	Present results of all investigations of possible causes of heterogeneity among study results.	NA
Reporting biases	21	Present assessments of risk of bias due to missing results (arising from reporting biases) for each synthesis assessed.	NA
Certainty of evidence	22	Present assessments of certainty (or confidence) in the body of evidence for each outcome assessed.	NA
**DISCUSSION**			
Discussion	23a	Provide a general interpretation of the results in the context of other evidence.	08-Nov
	23b	Discuss any limitations of the evidence included in the review.	10-Nov
	23c	Discuss any limitations of the review processes used.	11
	23d	Discuss implications of the results for practice, policy, and future research.	11
OTHER INFORMATION
Registration and protocol	24a	Provide registration information for the review, including register name and registration number, or state that the review was not registered.	4
	24b	Indicate where the review protocol can be accessed, or state that a protocol was not prepared.	4
	24c	Describe and explain any amendments to information provided at registration or in the protocol.	NA
Support	25	Describe sources of financial or non-financial support for the review, and the role of the funders or sponsors in the review.	1
Competing interests	26	Declare any competing interests of review authors.	1
Availability of data, code and other materials	27	Report which of the following are publicly available and where they can be found: template data collection forms; data extracted from included studies; data used for all analyses; analytic code; any other materials used in the review.	NA

From: Page MJ, McKenzie JE, Bossuyt PM, Boutron I, Hoffmann TC, Mulrow CD, et al. The PRISMA 2020 statement: an updated guideline for reporting systematic reviews. BMJ 2021;372:n71. doi: 10.1136/bmj.n71

For more information, visit: http://www.prisma-statement.org/

### Search Strategy

09 April 2020 PUBMED

MeSH terms + Synonyms

“Brazil” AND (“Cytochrome P-450 21-Hydroxylase” OR “Cytochrome P 450 21 Hydroxylase” OR “Cytochrome P-450(c-21) ” OR “Progesterone 21-Hydroxylase” OR “Progesterone 21 Hydroxylase” OR “Cytochrome P-450 CYP21” OR “Cytochrome P 450 CYP21” OR “Steroid 21-Monooxygenase” OR “Steroid 21 Monooxygenase” OR “Cytochrome P450c21” OR “Cytochrome P-450 c21” OR “Cytochrome P 450 c21” OR “P-450 c21, Cytochrome” OR “21-Hydroxylase” OR “21 Hydroxylase” OR “Steroid-21-Hydroxylase” OR “Steroid 21 Hydroxylase” OR “Steroid 21-Hydroxylase” OR “Cytochrome P450 Family 21” OR “CYP21 Enzymes” OR “CYP21 Family” OR “Congenital Adrenal Hyperplasia” OR “Adrenal Hyperplasias, Congenital” OR “Congenital Adrenal Hyperplasias” OR “Hyperplasias, Congenital Adrenal” OR “Hyperplasia, Congenital Adrenal” OR “Adrenal Hyperplasia, Congenital”)

157 records.

Embase

Emtree + Synonym

(“CYP21” OR “cytochrome P 450 21” OR “cytochrome p 450 c21” OR “cytochrome P 450 CYP21” OR “cytochrome P 450 family 21” OR “cytochrome P-450 CYP21” OR “cytochrome P- 450 family 21” OR “cytochrome P450 21” OR “cytochrome p450 c21” OR “cytochrome P450 CYP21” OR “p450 (c 21)” OR “p450 c21” OR “cytochrome P450 family 21” OR “21 hydroxylase” OR “21 hydroxylation” OR “21 steroid hydroxylase” OR “21alpha hydroxylase” OR “21beta hydroxylase” OR “cytochrome P 450 21 hydroxylase” OR “cytochrome P450 21 hydroxylase” OR “e.c. 1.14.1.8” OR “e.c. 1.14.99.10” OR “steroid 21 hydroxylase” OR “steroid 21 hydroxylation” OR “steroid 21-hydroxylase” OR “steroid 21-monooxygenase” OR “steroid c 21 hydroxylase” OR “steroid, hydrogen donor oxygen:oxidoreductase (21 hydroxylating)” OR “steroid 21 monooxygenase” OR “21 hydroxylase deficiency syndrome” OR “adrenal hyperplasia type 3” OR “adrenal hyperplasia, congenital” OR “adrenal hyperplasia, congenital” OR “adrenal hyperplasia, congenital lipoid” OR “adreno genital syndrome” OR “adrenogenital syndrome” OR “adrenogenital syndrome congenital” OR “adrenogenital syndrome type 3” OR “adrenogenital syndrome, congenital” OR “androgenital syndrome” OR “cah 1” OR “cholesterol 20, 22 desmolase deficiency syndrome” OR “cholesterol monooxygenase (side chain cleaving) deficiency syndrome” OR “cholesterol side chain cleavage enzyme deficiency syndrome” OR “congenital adrenal gland hyperplasia” OR “congenital adrenal gland lipoid hyperplasia” OR “congenital adrenal hyperplasia 1” OR “congenital adrenal hyperplasia type 1” OR “congenital adrenal lipoid hyperplasia” OR “congenital adrenal virilism” OR “congenital adrenal virilizing hyperplasia” OR “congenital adrenocortical hyperplasia” OR “congenital adrenogenital syndrome” OR “congenital lipoid adrenal gland hyperplasia” OR “congenital lipoid adrenal hyperplasia” OR “debre fibiger syndrome” OR “mckusick 20191” OR “steroid 21 monooxygenase deficiency syndrome” OR “congenital adrenal hyperplasia”) AND (“Federative Republic of Brazil” OR “United States of Brazil” OR “Brazil”)

Filters: Embase and Embase+Medline 335 records

LILACS

Title, abstract, subject

(“Cytochrome P450 Family 21” OR “CYP21 Enzymes” OR “CYP21 Family” OR “21 Hydroxylase” OR “Steroid 21-Hydroxylase” OR “21-Hydroxylase” OR “Cytochrome P 450 21 Hydroxylase” OR “Cytochrome P 450 CYP21” OR “Cytochrome P 450 c21” OR “Cytochrome P-450 21-Hydroxylase” OR “Cytochrome P-450 CYP21” OR “Cytochrome P-450 c21” OR “Cytochrome P-450(c-21)” OR “Cytochrome P450c21” OR “P- 450 c21, Cytochrome” OR “Progesterone 21 Hydroxylase” OR “Progesterone 21-Hydroxylase” OR “Steroid 21 Hydroxylase” OR “Steroid 21 Monooxygenase” OR “Steroid 21-Monooxygenase” OR “Steroid-21-Hydroxylase” OR “Adrenal Hyperplasia, Congenital” OR “Adrenal Hyperplasias, Congenital” OR “Congenital Adrenal Hyperplasia” OR “Congenital Adrenal Hyperplasias” OR “Hyperplasia, Congenital Adrenal” OR “Hyperplasias, Congenital Adrenal”) AND (Brazil)

Filter: LILACS 28 records BDTD

(Todos os campos: Hiperplasia Suprarrenal Congênita OU Todos os campos: Hiperplasia Adrenal Congênita OU Todos os campos: Família 21 do Citocromo p450 OU Todos os campos: 21-Hidroxilase OU Todos os campos: Enzimas CYP21 OU Todos os campos: Família CYP21)

328 records SciELO

((CYP21 Enzymes) OR (CYP21 Family) OR (Cytochrome P450 Family 21) OR (21 Hydroxylase) OR

(21-Hydroxylase) OR (Cytochrome P 450 21 Hydroxylase) OR (Cytochrome P 450 CYP21) OR (Cytochrome P 450 c21) OR (Cytochrome P-450 21-Hydroxylase) OR (Cytochrome P-450 CYP21) OR (Cytochrome P-450 c21) OR (Cytochrome P-450(c-21)) OR (Cytochrome P450c21) OR (P-450 c21, Cytochrome) OR (Progesterone 21 Hydroxylase) OR (Progesterone 21-Hydroxylase) OR (Steroid 21 Hydroxylase) OR (Steroid 21 Monooxygenase) OR (Steroid 21-Monooxygenase) OR (Steroid-21-Hydroxylase) OR (Steroid 21-Hydroxylase) OR (Adrenal Hyperplasias, Congenital) OR (Congenital Adrenal Hyperplasia) OR (Congenital Adrenal Hyperplasias) OR (Hyperplasia, Congenital Adrenal) OR (Hyperplasias, Congenital Adrenal) OR (Adrenal Hyperplasia, Congenital))

Filter: Brasil 107 records

Total: 955 records

Other sources: 1

Search update (Apr 22)

The same protocol was used. A total of 77 records identified
